# Craft-and-Stick Xurographic Manufacturing of Integrated Microfluidic Electrochemical Sensing Platform

**DOI:** 10.3390/bios13040446

**Published:** 2023-03-31

**Authors:** Supatinee Kongkaew, Lingyin Meng, Warakorn Limbut, Guozhen Liu, Proespichaya Kanatharana, Panote Thavarungkul, Wing Cheung Mak

**Affiliations:** 1 Biosensors and Bioelectronics Centre, Division of Sensor and Actuator Systems, Department of Physics, Chemistry and Biology, Linköping University, SE-581 83 Linköping, Sweden; 2 Center of Excellence for Trace Analysis and Biosensor, Prince of Songkla University, Hat Yai 90112, Songkhla, Thailand; 3 Center of Excellence for Innovation in Chemistry, Faculty of Science, Prince of Songkla University, Hat Yai 90112, Songkhla, Thailand; 4 Division of Health and Applied Sciences, Faculty of Science, Prince of Songkla University, Hat Yai 90112, Songkhla, Thailand; 5 School of Medicine, The Chinese University of Hong Kong, Shenzhen 518172, China; 6 Division of Physical Science, Faculty of Science, Prince of Songkla University, Hat Yai 90112, Songkhla, Thailand; 7 Department of Biomedical Engineering, The Chinese University of Hong Kong, Hong Kong SAR, China

**Keywords:** graphene paper electrode, microfluidic device, biosensor, xurographic, craft-and-stick manufacturing

## Abstract

An innovative modular approach for facile design and construction of flexible microfluidic biosensor platforms based on a dry manufacturing “craft-and-stick” approach is developed. The design and fabrication of the flexible graphene paper electrode (GPE) unit and polyethylene tetraphthalate sheet (PET)6/adhesive fluidic unit are completed by an economic and generic xurographic craft approach. The GPE widths and the microfluidic channels can be constructed down to 300 μm and 200 μm, respectively. Both units were assembled by simple double-sided adhesive tapes into a microfluidic integrated GPE (MF-iGPE) that are flexible, thin (<0.5 mm), and lightweight (0.4 g). We further functionalized the iGPE with Prussian blue and glucose oxidase for the fabrication of MF-iGPE glucose biosensors. With a closed-channel PET fluidic pattern, the MF-iGPE glucose biosensors were packaged and sealed to protect the integrated device from moisture for storage and could easily open with scissors for sample loading. Our glucose biosensors showed 2 linear dynamic regions of 0.05–1.0 and 1.0–5.5 mmol L^−1^ glucose. The MF-iGPE showed good reproducibility for glucose detection (RSD < 6.1%, *n* = 6) and required only 10 μL of the analyte. This modular craft-and-stick manufacturing approach could potentially further develop along the concept of paper-crafted model assembly kits suitable for low-resource laboratories or classroom settings.

## 1. Introduction

The combination of microfluidics and biosensors provide an integrative platform for sample-to-answer biosensing applications. Microfluidic technology is important for sample loading and reagent transport, immobilization of biorecognition molecules, accommodation of bioreactions, and biosensing reactions at the sensor interface, particularly electrochemical transducer [1]. A well-known example of an integrated electrochemical biosensor is the blood glucose test strip comprised of a screen-printed electrode integrated with a capillary fluidic channel for the monitoring of glucose levels in diabetic patients creating the largest market in the field of commercial biosensors [2,3]. Measuring blood glucose is important for diabetes patients (~537 million diabetes patients worldwide) to monitor their blood glucose levels with disposable glucose biosensor strips. Beyond that, integrated microfluidic biosensors are useful in a wide range of applications, including clinical diagnostics [4], microbiology [5], and environmental monitoring [6]. Therefore, the development of an easy-to-implement and affordable method for the fabrication and assembly of integrated electrochemical microfluidics biosensors may encourage the future development of point-of-care (PoC) and point-of-use (PoU) biosensors to improve people’s life quality in developing countries where resources are limited.

For the fabrication of the microfluidic platform, various techniques have been developed, such as photolithography [7], 3D printing [8], CNC micro-milling [9], UV laser photoablation [10], and xurography [11]. Photolithography allows precise and high-resolution fabrication of polydimethylsiloxane (PDMS) based microfluidic devices, while it requires labor-intensive fabrication steps and a specially equipped light-controlled environment. Recently, 3D printing is an emerging technology for the fabrication of complex 3D polymer-based microfluidic devices via computer aid modeling [12,13], while the resolution of 3D printing requires further improvement to catch up with photolithography. CNC micro-milling and laser photoablation are top-down approaches by removing the substrate materials for the fabrication of microfluidic patterns [14]. The vigorous milling and high-energy ablation process are effective for the manufacture of fluidic devices with robust materials such as glass [15,16], silicon [17], and polymers (i.e., PDMS, PMMA) [18,19], while the process may create surface defects for temperature sensitive, thin and fragile substrates. Laser processing, including CO_2_ lasers and UV lasers, uses high energy to craft and create patterns on various hard substrates such as glass, borosilicate, silica, and quartz [20], and a few temperature-resisted polymer substrates [21,22]. However, the high-energy laser cutting process may not be suitable for temperature-sensitive polymer substrates or fire-catching cellulose or paper substrates resulting in a poor quality final product due to the melting, burning, and vaporization of substrate materials [23,24]. While xurography is a complementary approach using computer-controlled knife crafting for the creation of microfluidic patterns onto thin, flexible, and soft substrate materials such as papers and polymer films into various designed patterns as a universal technique for various platforms.

For an integrated electrochemical microfluidic biosensor, a suitable and complementary method for the fabrication of the electrode component is another important consideration. Various approaches have been used for the fabrication of affordable and disposable electrodes, such as pencil drawing [25,26], stencil printing [27], screen printing [28], and inkjet printing [29]. Among all, printing/deposition of conductive carbon or metallic inks onto a substrate material is the most common approach for the fabrication of printed electrodes. For the screen/stencil printing approach, it required an additional tailor-designed mask to guide the deposition of conductive inks into electrode patterns, followed by a high-temperature curing process. The screen printing approach allows high throughput fabrication of printed electrodes, while the technique is not convenient for changing electrode designs that require the redesign and fabrication of a new mask. In contrast, inkjet printing allows a more flexible design and fabrication of printed electrodes, while it requires a more precise ink formulation to prevent the clogging of the nozzle during the inkjet printing process and a specific solvent to support a faster evaporation and drying process [30]. For the development of integrated electrochemical microfluidic biosensors, a combination of the above individual methods for the fabrication of the microfluidic unit and the electrode unit is typically used. While the integration and assembly of electrochemical microfluidic biosensors with radically different fabrication methods and multiple instrumentations remain a technical challenge. Recently, a new demand for thin, light-weight and flexible features of microfluidic biosensors for emerging applications such as wearable biosensors [31], attachable/mountable biosensors [32], and stretchable biosensors [33] requires novel fabrication and integration approaches for the development of flexible microfluidic biosensors. Graphene paper is a novel form of material supported by graphene nanosheets, which has attracted much attention in emerging electronic and bioelectronic devices because of its processability, flexibility, lightness, and high conductivity [34]. The applications of graphene paper in a variety of fields, such as energy storage [35] and flexible electronics [36], remain in the initial stages. The integration of graphene paper electrodes with flexible microfluidic combined with a facile fabrication approach could support the future development of new flexible microfluidic integrated graphene paper-based sensors and biosensors.

Here, we demonstrate an innovative modular approach for the dry manufacturing of flexible microfluidic biosensor platforms. The design and fabrication of the flexible graphene paper electrode units, polyethylene tetraphthalate sheet (PET) fluidic units, and adhesive units are completed by a facile and generic xurographic crafting approach. The resolution of the conductive tracks prepared by the xurographic method on the graphene paper and fluidic channel on the PET substrates were evaluated. The electrochemical characteristics and morphology of the crafted GPE were characterized. The GPE units and PET/adhesive fluidic units were fabricated and assembled via a craft-and-stick approach by simple double-sided adhesive tapes into a microfluidic integrated GPE (MF-iGPE) that are flexible, thin (<0.5 mm) and lightweight (0.4 g). We further functionalized the GPE with Prussian blue (PB) and glucose oxidase (GOx) for the fabrication of MF-iGPE glucose biosensors. With a closed-channel PET fluidic pattern, the MF-iGPE glucose biosensors were packaged and sealed to protect them from moisture for storage. For measurement, both ends of the sealed fluidic channel were opened with scissors, and samples were loaded into the MF-iGPE by capillary flow.

## 2. Materials and Methods

### 2.1. Materials

Graphene paper, potassium hexacyanoferrate (III), potassium hexacyanoferrate (II) trihydrate, iron (III) chloride, potassium chloride, hydrochloric acid, human serum, and Gox (from Aspergillus niger, 145,200 unit/g) were obtained from Sigma-Aldrich (St. Louis, MO, USA). Disodium hydrogen phosphate, potassium dihydrogen phosphate, glucose, and chitosan were purchased from Merck (Darmstadt, Germany). PET sheets and double-sided adhesive tape were purchased from Biltema (Linköping, Sweden). An Ag/AgCl ink (DuPontTM 5874) was obtained from Dupont Ltd. (Stevenage, U.K.). All chemicals were of analytical reagent grade. The phosphate buffer solution (PBS, 0.1 mol L^−1^, pH 7.4) was prepared by mixing a stock solution of potassium dihydrogen phosphate and disodium hydrogen phosphate. All solutions were prepared using deionized water (Milli-Q purification system, MerckMillpore, MA, USA).

### 2.2. Design and Fabrication of Patterned GPE

The GPEs were fabricated via the xurographic technique using a robotic cutting device (Brother ScanNcut, model number CM900). Firstly, the electrode patterns were designed using the Brother Canvas workplace program version 2.2.0. A graphene paper was attached to a PET sheet with double-sided adhesive tape. The width parameter of the GPE conductive track was investigated with cutting resolutions between 0.2 and 1.0 mm and a fixed length of 15 mm, with a square-sharped connection terminal of 5 × 5 mm and a circular-shape electrode terminal of 3 mm diameter. The blade height of the robotic cutting instrument was optimized to the number 3 setting for crafting through the graphene paper and double-sided adhesive tape while keeping the PET substrates intact. After the crafting process, the patterned GPE supported with PET was obtained by peeling off the excess graphene paper. The obtained GPE was treated with oxygen plasma treatment (Diener electronic, Plasma Surface Technology, Pico, Germany) for 1 min. An integrated GPE (iGPE) module consists of a working electrode, a solid-state Ag/AgCl reference electrode (replacing the conventional glass Ag/AgCl reference electrode), and an auxiliary electrode. The solid-state reference electrode was constructed by painting/casting of Ag/AgCl-based ink on the RE location of the conductive track, followed by curing it for 5 min on a hotplate at 120 °C. Finally, an adhesive tape (5.0 × 10.0 mm) was attached to the top of the conductive track of the iGPE as an insulating layer to define the working area of the electrode and isolate the conductive track from the solution. The resistance of the conductive track of the GPE was measured with a digital multimeter (DMM 220 model P06231411, Multimetrix, Asnières-Sur-Seine, France). To improve the electrochemical properties of GPE, it was treated with oxygen plasma and stored in a sealed box until usage [37].

### 2.3. Design and Fabrication of the PET/Adhesive Fluidic Unit

The fluidic units were fabricated via xurographic technique using the robotic cutting device and the pattern of the fluidic channel was designed by the Brother Canvas workplace program. The fluidic unit was designed with an inlet channel (2 × 10 mm), an outlet channel (2 × 10 mm), and an ellipse-shaped measuring chamber (9 × 7.5 mm) that serves as a sample reservoir to be mounted on the GPE sensor area. Three layers of double-sided adhesive tape with a total height of ~0.2 mm were attached to a PET sheet. The blade height of the robotic cutting instrument was optimized to the number 5 setting for crafting through the double-sided adhesive tape while keeping the PET substrates intact. The negative pattern of the adhesive tape was peeled off and formed the fluidic pattern, and finally sealed with a PET top layer to create the PET/adhesive fluidic unit. The assembled PET/adhesive fluidic units were loaded/immersed with a dye solution (phenol red) for leakage examination.

### 2.4. Functionalization of GPE with PB and GOx

PB was deposited onto the GPE by applying a constant potential at 0.4 V for 300 s in 1 mol L^−1^ KCl and 3 mmol L^−1^ HCl containing a mixture of 5 mmol L^−1^ K_3_FeCN_6_ and FeCl_3_. The PB-modified GPE (PB-GPE) was activated in a 0.1 mol L^−1^ KCl and 0.01 mol L^−1^ HCl solution by cycling at the potential range between −0.2 and 0.6 V with a scan rate of 0.05 V s^−1^ for 10 cycles. Then, 3 µL of GOx solution in 0.1 mol L^−1^ PBS (10 mg mL^−1^) equivalent to 4.36 enzyme units was deposited on the PB-GPE and dried at 4 ℃ for 2 h, followed by the addition of 4 µL chitosan solution (1%, in 1% acetic acid).

### 2.5. Assembling of the Microfluidic-Integrated Graphene Paper Electrode (MF-iGPE)

The MF-iGPE was assembled via a simple sticking approach. Firstly, the PET-supported iGPE module consisted of the crafted electrode-pattern was placed in an upright position with the electrode facing upward, while the PET/adhesive fluidic unit consisted of the crafted fluidic-patterned and open-structured measuring chamber with the adhesive layer facing downward. Then, the working, reference, and counter electrodes of the iGPE unit were aligned with the ellipse-shaped measuring chamber of the PET/adhesive fluidic unit, followed by pressing together the iGPE unit and PET/adhesive fluidic unit forming the MF-iGPE biosensor held by the negative adhesive layer.

### 2.6. Characterizations and Electrochemical Measurements

The morphology and structure of the GPE were characterized by a scanning electron microscope (SEM, LEO 155 Gemini, Zeiss, Germany) and energydispersive X-ray spectroscopy (EDS, Oxford Instruments, Abingdon, UK). Raman spectra were acquired with a LabRAM HR 800 Raman spectrometer (Horiba Jobin Yvon, Palaiseau, France) using a 660 nm laser with a power of 5 mW. The electrochemical measurements were performed by a BiPotentiostat/galvanostat μStat400 (Metrohm DropSens, S.L., Asturias, Spain). The electrochemical properties of the electrodes were characterized in 0.1 mol L^−1^ KCl containing 2 mmol L^−1^ [Fe(CN)_6_]^3−/4−^ and through cyclic voltammetry. Serum samples were obtained from a diabetes patient at Hatyai Hospital, Hat Yai, Songkhla, Thailand.

## 3. Results and Discussion

### 3.1. Generic Fabrication of Flexible Electrode and Fluidic Units via Xurography

**Figure 1:**
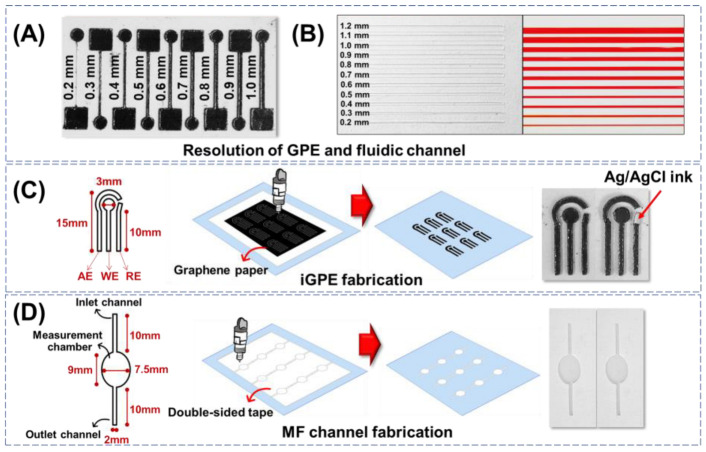
(**A**) Resolution of graphene paper conductive tracks fabricated via xurography. (**B**) Resolution of fluidic channels fabricated via xurography. (**C**) The design and fabrication of the iGPE module consisted of AE: auxiliary electrode, WE: working electrode, and RE: reference electrode. (**D**) The design and fabrication of the fluidic module consisted of inlet and outlet channels and an ellipse-shaped measuring chamber.

The minimum resolution of the xurographic fabrication process for the electrode and fluidic modules was evaluated. Firstly, the resolution of the conductive tracks on GPE fabricated via xurography was evaluated. The cutting edges of the GPE are relatively smooth, with intact conductive tracks from 0.3 to 1.0 mm, as observed in Figure 1A. While a disconnected conductive track was observed with cutting resolution further reduced to 0.2 mm. The resistance of the conductive tracks was then evaluated by a two-probe multimeter. The resistance decreased from 5.5 Ω to 2.0 Ω by increasing the width of the electrode track from 0.3 to 1.0 mm. The results are consistent with the relationship between the electrical resistance and different sizes of electrode width as described in the reported literature [38]. Furthermore, the circular shape of the 3 mm working electrodes connected by the 0.3-, 0.5-, and 1-mm conductive tracks were evaluated with a ferrocyanide probe for electrochemical characterizations. There was no significant difference in the electrochemical characteristics of the working electrodes with varying widths of the conductive tracks indicated by the peak current and peak-to-peak separation (∆Ep) (Figure S1A–C in Supplementary Materials). Thus, the xurographic method allows the crafting of conductive features on a graphene paper substrate with a minimum resolution of 0.3 mm, which provides a good electrochemical characteristic with less affected by the conductive tracks.

The resolution of the xurographic method to fabricate the PET/adhesive fluidic channels with different widths ranging from 0.2 to 1.2 mm was evaluated (Figure 1B, left). The edge of the channel appeared smooth, and the width of the fluidic channel is defined by the resolution distance between the two adjunct cuts as low as 0.2 mm width prepared by a simple home-used xurography machine. To investigate the possible fabrication of the fluidic channel, the open PET/adhesive fluidic channels were then covered with a top layer of PET via the negative adhesive to create a sealed PET/adhesive/PET fluidic unit. The assembled PET/adhesive/PET fluidic units were examined using a dye solution (phenol red) for capillary loading and leakage examination. Figure 1B shows the dye solution was able to load via capillary flow into all the fluidic channels with different widths and without observable leakage. The results indicated that both electrode and fluidic modules built with graphene paper and double-sided adhesive tape could be completed using only one instrument, the xurographic instrument.

Next, the fabrication of a three-electrode integrated GPE (iGPE) consisting of a working electrode, an auxiliary electrode, and a reference electrode was designed, as illustrated in Figure 1C. With the current design pattern of iGPE, a standard A4-sized graphene paper (~44.81 EUR/sheet) could accommodate the fabrication of up to 594 pieces of iGPE (EUR ~0.08 per iGPE) using a low-cost xurographic machine (EUR ~500) that provides a highly facile and affordable dry-manufacturing approach for the fabrication of a thin, flexible and light-weight iGPE for the subsequential integration of the fluidic unit. The design and fabrication of the PET/adhesive fluidic unit are illustrated in Figure 1D. After the xurographic process, the grafted adhesive was removed, creating the open-structured features, while the remaining adhesive allowed the simple attachment of the PET/adhesive fluidic unit with the PET-supported GPE. Nevertheless, the resolution of the conductive features created on graphene paper and the fluidic channel features on PET/adhesive substrate are currently demonstrated by a simple, low-cost, home-used xurographic machine, and the resolutions of the fabrication methods could further be improved by using an advanced high-resolution xurographic instrument.

### 3.2. Morphological and Electrochemical Characterizations of the GPE

**Figure 2:**
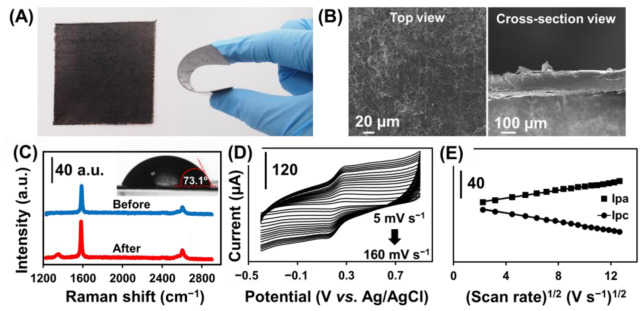
(**A**) Photograph of GPE and the bending of GPE attached to the PET sheet. (**B**) SEM image of GPE at the top view (left) and cross-sectional (right) view. (**C**) Raman of GPE before and after plasma treatment and contact angle of GPE after plasma treatment (Inset). (**D**) Cyclic voltammograms with different scan rates from 5 to 160 mV s^−1^ in 0.1 mol L^−1^ KCl containing 2 mmol L^−1^ [Fe(CN)_6_]^3−/4−^ at GPE. (**E**) Plots of the Ipa and Ipc versus the square root of scan rates (from 5 to 160 mV s^−1^) at GPE.

Figure 2A shows the physical appearance and bending flexibility of the GPE. The GPE shows a wrinkle-structured surface morphology composed of randomly stacked graphene nanosheets (Figure 2B, left) and with a thickness of approximately 150 μm estimated by the cross-sectional SEM image (Figure 2B, right). The Raman spectrum (Figure 2C) evaluates the structural properties of the GPE before (blue line) and after (red line) oxygen plasma treatment. Two main characteristic bands are representing the specific band for graphite-like materials with sp2 carbon, including the G band (∼1584 cm^−1^) and the 2D band (∼2654 cm^−1^) corresponding to the crystalline ordering of the graphitic basal plane and the stacking order, respectively [39,40]. After treating GPE with oxygen plasma, another new band at ∼1341 cm^−1^ (D band) is ascribed to the structural disorder and defect of carbon material [41], while the D band was absent in the non-treated GPE. The ID/IG ratio is used to characterize the level of disorder in the carbon structure [40]. The treated GPE has an ID/IG ratio of 0.096, implying a higher disorder and defects in the graphene structure contributed by the oxygenated functional group [42], which is further verified by the increased surface hydrophilicity with the decrease in contact angle from 90.26° to 73.05° (Figure 2C inset).

The electrochemical kinetics of the GPE was investigated by studying the effect on the scan rate from 5 to 160 mV s^−1^ in a 0.1 mol L^−1^ KCl solution containing 2 mmol L^−1^ [Fe(CN)_6_]^3−/4−^ using a cyclic voltammetry technique (Figure 2D). The anodic (Ipa) and cathodic (Ipc) peak currents increased as the scan rate increased. The linear regression equations were Ipa = (2.77 ± 0.04) v^1/2^ − (0.5 ± 0.4), R^2^= 0.9960 and Ipc = −(3.04 ± 0.03)x + (1.4 ± 0.3), R^2^ = 0.9981, respectively (Figure 2E). The current response increased linearly versus the square root of the scan rate from 5 to 160 V s^−1^, suggesting the electrochemical kinetic is a diffusion-controlled process. The apparent diffusion coefficient of [Fe(CN)_6_]^3−/4−^ on the GPE was calculated to be 2.1 × 10^−5^ cm^2^ s^−1^ using the Randles–Sevcik equation.

### 3.3. Integration and Characterization of the MF-iGPE Platform

**Figure 3:**
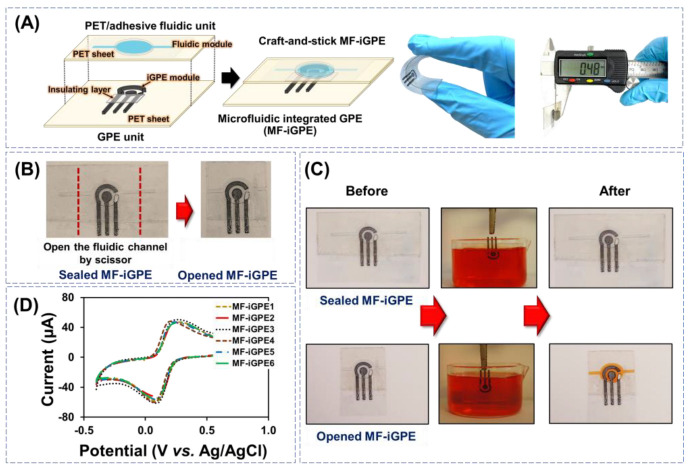
(**A**) Schematic of craft-and-stick MF-iGPE. (**B**) Photograph of sealed MF-iGPE devices and the opened MF-iGPE. (**C**) The photograph of sealed and opened MF-iGPE before and after incubation in the dye solution. (**D**) Cyclic voltammograms of redox solution obtained by different MF-iGPE platforms.

The iGPE and fluidic modules were assembled manually via a facile craft-and-stick approach for the fabrication of MF-iGPE, as shown in Figure 3A. The MF-iGPE is flexible, thin (~0.48 mm), and lightweight (0.4 g). With a closed-channel PET fluidic pattern, the MF-iGPE was packaged and sealed to minimize the influence of environmental factors such as humidity and physical contamination (Figure 3B, left). The fluidic channels can be easily opened by scissors, where a sample can be loaded into the opened MF-iGPE via a capillary flow (Figure 3B, right). To examine the device sealing and loading properties, the sealed MF-iGPE and opened MF-iGPE were fully immersed into a phenol red dye solution for 5 min and lifted for examination (Figure 3C). The results showed the sealed MF-iGPE remains dry and intact without observable contamination by the dye solution neither inside the fluidic module nor the iGPE surface. It demonstrated the built-in sealing of the MF-iGPE provides a facile and economic device packing solution to minimize contamination and facilitates the transport and storage of the MF-iGPE for distributed biosensors. After cutting the sealed ends, the opened fluidic channel of the MF-iGPE was effectively filled by the dye solution covering the iGPE sensor surface without leakage. The reproducibility of the MF-iGPE fabrication process was evaluated by studying six different MF-iGPE platforms with cyclic voltammetry using a 5 mmol L^−1^ [Fe(CN)_6_]^3−/4−^ in 0.1 mol L^−1^ KCl as shown in Figure 3D. The relative standard deviation (RSD) values of the cyclic voltammetry measurements for the Ipa and the Ipc were 2.2% and 5.9% (n = 6), respectively. The relatively small RSD value demonstrated good fabrication and operational reproducibility of the MF-iGPE devices.

### 3.4. Preparation and Characterization of the MF-iGPE Glucose Biosensor

**Figure 4:**
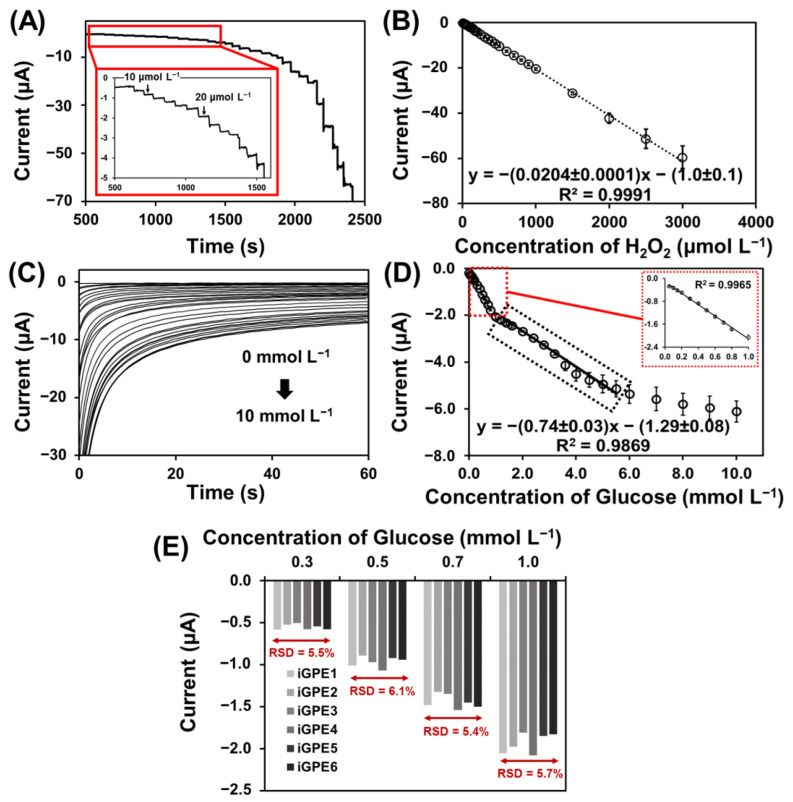
(**A**) Amperograms of PB/iGPE carried in 0.1 mol L^−1^ PBS pH 7.4 containing 0.1 mol L^−1^ KCl upon continuous injection of a standard solution of H_2_O_2_ (from 0.01 to 3.00 mmol L^−1^) at an applied potential of 0.0 V. (**B**) Linear range of H_2_O_2_ at PB/iGPE obtained from amperograms in the concentration range from 0.01 to 3.00 mmol L^−1^. (**C**) Chronoamperograms toward glucose concentration range from 0.0 to 10 mmol L^−1^ at GOx-PB/iGPE in 0.05 mol L^−1^ PBS containing 0.10 mol L^−1^ KCl at working potential of −0.05 V. (**D**) Calibration curve toward glucose in a concentration range from 0.05 to 1.0 and from 1.0 to 5.5 mmol L^−1^ recorded at 50 s. (**E**) Reproducibility of MF−iGPE toward different concentrations of glucose.

To establish efficient signal transduction in biosensing, PB was deposited on the iGPE for the reduction of an enzymatic intermediate H_2_O_2_ for the further development of GOx-based glucose biosensors. The PB-modified iGPE (PB/iGPE) was examined by SEM and EDX. Figure S2A,B shows a layer of nanocubic-structured PB on the iGPE surface [43]. Moreover, the appearance of the signature Fe, N, and K elements present in the EDX spectrum (Figure S2C) verified the successful preparation of the PB/iGPE. The electrochemical properties of PB/iGPE in the presence of an H_2_O_2_ solution were investigated using cyclic voltammetry (Figure S3). The results showed that in the presence of H_2_O_2_, the reduction peak of PB increased while the oxidation peak of PB decreased. Therefore, the PB reduction peak (at the potential of 0.05 V) was chosen as the operating potential for the next amperometric detection of glucose. Following that, the measurement of H_2_O_2_ based on PB/iGPE was carried out with the successive addition of H_2_O_2_ in the concentration range of 10–3000 µmol L^−1^ (0.1 mol L^−1^ PBS, 0.1 mol L^−1^ KCl) (Figure 4A). A good linear relationship between the current responses and the concentration range of H_2_O_2_ with a high sensitivity of 291.42 µA mmol^−1^ L cm^−2^ and a good linear correlation coefficient (R^2^ = 0.9991) was obtained (Figure 4B). Then, GOx was immobilized on the PB/iGPE to create GOx-PB/iGPE. Figure 4C presented that the chronoamperometric response of GOx-PB/iGPE easily reaches a steady state at a low concentration of glucose. At high concentrations of glucose, the current slowly reaches the steady-state. Figure 4D shows a calibration plot, which displayed 2 linear dynamic regions, from 0.05 to 1.0 mmol L^−1^ for the first region and from 1.0 to 5.5 mmol L^−1^ for the second region, respectively. The linear regression equations were y = −(1.98 ± 0.04)x − (0.12 ± 0.02), (R^2^ = 0.9965) for the first linear region and y = −(0.74 ± 0.03)x − (1.29± 0.08), (R^2^ = 0.9869) for the second linear region, respectively. This result of two linear ranges was similar to that reported in earlier publications using glucose oxidase for glucose detection [44,45,46].

Shaolin et al. suggested that the catalytic reaction of immobilized enzymes takes place predominantly on the electrode surface in the low concentration (first-order reaction). However, the reaction on the electrode surface and the diffusion occurred simultaneously at high glucose concentrations. As a result of the response time delays, and slowly reach a steady-state at high glucose concentration (transition from first to zero order) [47]. Moreover, at high glucose concentrations, the linear range of the enzymatic glucose biosensor could be limited by the Michaelis constant (Km) of GOx [48], diffusion effect, saturation, and rate of glucose adsorption [49]. It should be noted that the linearity was shorter compared to the detection of H_2_O_2_ since the enzymatic detection of glucose was based on the catalytic of H_2_O_2_ reduction mediated by PB. The linear range can be further extended in several ways such as; (1) using the additional diffusion layer [50]; (2) using protein-engineered enzymes with increased K_m_ value [51]; (3) the use of nanoparticles for increased enzyme loading [52]. The sensitivity of the glucose biosensor is 28.3 μA mmol^−1^ L cm^−2^, and the limit of detection is 31 μmol L^−1^ glucose (3σ/sensitivity). The reproducibility of the iGPE glucose biosensor was studied by a chronoamperometry technique. Six different iGPE glucose biosensors were tested by measuring different glucose concentrations, 0.3, 0.5, 0.7, and 1.0 mmol L^−1^ (Figure 4E). The RSD values are 5.5, 6.1, 5.4, and 5.7%, for 0.3, 0.5, 0.7, and 1.0 mmol L^−1^ glucose, respectively. According to the AOAC Official Methods of Analysis Guideline, the predicted relative standard deviation of reproducibility for analyte concentration below 100 ppm (equivalent to between 0.3 and 0.5 mmol L^−1^ glucose) should be less than 8% and for analyte concentration above 100 ppm or 0.1% (equivalent to between 0.7 and 1.0 mmol L^−1^ glucose) should be less than 6%. Our RSD values for different glucose concentrations were from 5.5 to 6.1%, and from 5.4 to 5.7% for concentrations from 0.3 to 0.5 mmol L^−1^ and concentrations from 0.7 to 1.0 mmol L^−1^, respectively. Our measured results were below the AOAC guideline RSD values indicating a good reproducibility [53]. Furthermore, we evaluated the selectivity of the iGPE glucose biosensor with common interference in physiological fluidics, such as ascorbic acid and dopamine [54], using the chronoamperometry technique (Figure S4A,B). The interfering substances should have less than 10% of glucose response in the sample, according to the ISO 15,197 criteria [55]. As a control sample, the normal glucose concentration of 5.0 mmol L^−1^ glucose was investigated, and the response signal was normalized to 100% (Figure S4C). Figure S4C shows the current response of glucose changed by less than 10% in the presence of 10 μmol L^−1^ ascorbic acid and 2.5 μmol L^−1^ dopamine indicating a good selectivity of the iGPE glucose biosensor to be applied for the detection of glucose in perspiration [50] and diluted serum sample [54]. The stability of Gox-PB/iGPE was investigated with electrodes stored at 4 °C and measured the current responses of 5.0 mmol L^−1^ glucose on days 1, 5, and 10, respectively. At 10 days, the current response retains 97% of its initial value (Figure S5A), demonstrating a good storage stability. The repeatability of the proposed glucose biosensor was demonstrated in Figure S5B using the current response of 5.0 mmol L^−1^ glucose. The RSD value for repeated measurements was 9.7% (*n* = 14). In addition, the fabrication methods to prepare the sensors electrodes and microfluidic units, and the analytical performances of iGPE glucose biosensors were included in Table 1. Our proposed method provided good sensitivity of 28.3 μA mmol^−1^ L cm^−2^ (first linear region) and a low limit of detection of 0.031 mmol L^−1^ glucose (3σ/sensitivity), which is comparable to other reported platforms. In this work, we developed an innovative a dry manufacturing approach for the design and assembly (craft-and-stick) of a disposable integrated microfluidic biosensor platform prepared solely with an economic home-used cutting machine (we use the Brother ScanNcut CM900 that cost about 500 Euro). The low-cost and facile craft-and-stick approach is similar to the “paper-crafted model” concept with modules designed to build an integrated microfluidic electrochemical sensor platform. This approach is innovative and advantageous for developing a disposable microfluidic sensor platform. The design of the sensor electrodes and microfluidic channels with the crafted graphene paper (e.g., configuration and dimension) and crafted PET substrate are easily tailored with a new drawing using a generic xurographic method. In contrast to other report methods, tailoring electrode design using the screen printing method (wet-chemistry) would require fabricating a new screen, and tailoring PDMS microfluidic would require a new mask prepared by a photolithograph.

### 3.5. Practical Application of MF−iGPE Glucose Biosensor for Serum Sample

The practical application of the MF-iGPE glucose biosensor was demonstrated by the measurement of glucose in serum sample. A standard glucose solution calibration curve was constructed and used to calculate the glucose concentration in real serum samples. Samples were diluted ten times with PBS before measurement. The measured serum glucose concentration by the MF-iGPE was validated and compared by the standard hexokinase-spectrophotometric method (Table 2). The glucose level of a diabetic patient and a normal subject measured by the MF-iGPE glucose biosensor was 9.6 ± 1.3 mmol L^−1^ and 6.8 ± 0.8 mmol L^−1^ (*n* = 3, n representing the number of MF-iGPE platforms), respectively. The measured values were in close agreement with the values obtained by the standard method and the literature [66,67], suggesting the potential application for PoC detection of glucose levels in real serum samples. Furthermore, the accuracy of the proposed device was studied by spiking standard glucose concentrations of 1.0, 2.0, and 3.0 mmol L^−1^ in the human serum sample with a satisfactory recovery within the range of 88.2–108.8%. (Table S1). The MF-iGPE platform, in combination with the advantages of easy implementation and low cost, would support the future development of PoC and PoU biosensors to improve the quality of life for people in developing countries where resources are limited.

## 4. Conclusions

We developed an innovative dry manufacturing approach for the design and assembly (craft-and-stick) of a disposable integrated microfluidic biosensor platform prepared solely with an economical home-used cutting machine. The low-cost and facile craft-and-stick approach is similar to the “paper−crafted−model” concept, with modules designed to build an integrated microfluidic electrochemical sensor platform. It is innovative and advantageous for developing a disposable microfluidic sensor platform. The design of the sensor electrodes and microfluidic channels with the crafted graphene paper (e.g., configuration and dimension) and crafted PET substrates are easily tailored with a new drawing using a generic xerographic method. The fabrication process is fast, low-cost material (EUR 0.08 per device), and easy without the need for special equipment or clean rooms. The iGPE was successfully fabricated for electrochemical analysis of the redox probe solution investigated by the cyclic voltammetry technique (RSD < 5.9%). Prussian blue (PB) and glucose oxidase (GOx) were further modified on iGPE for the fabrication of MF−iGPE glucose biosensors, which provided 2 linear dynamic regions of 0.05–1.0 mmol L^−1^ and 1.0−5.5 mmol L^−1^ glucose. With the closed channel of MF-iGPE glucose biosensors, our device would be protected against humidity. The sealed MF-iGPE would easily open with scissors to load the sample for detection of glucose concentration. The development of an easy-to-implement and affordable method for the fabrication and assembly of integrated electrochemical microfluidics biosensors may encourage the future development of point-of-care and point-of-use biosensors to improve people’s life quality in developing countries where resources are limited.

## Figures and Tables

**Table 1:** The analytical performance of disposable electrode corporate with and without microfluidic by various procedures for glucose biosensors.

Electrode Fabrications		Microfluidic Fabrications						Performances					
Substrates	Techniques	Materials	Techniques	Sample Volume (µL)	Integrate AE and RE	Cost/Device	Built−In Packaging	Modified Electrodes	Techniques	Linear (mmol L^−1^)	Sensitivity	LOD (mmol L^−1^)	Ref.
Graphene paper	Xurography	PET	Xurography	10.0	/	EUR 0.08	/	GOx−Chi−PB/iGPE	Chrono (E = −0.05 V)	0.05−1.0, 1.0−5.5	28.3 μA mmol^−1^ L cm^−2^	0.031	This work
Paper	Screen printing	Paper	Wax printing	−	/	−	N/A	GOx/Chi/Naf/PB ink	Amp (E = −0.1 V)	0−1.9	35.7 μA mmol^−1^ L cm^−2^	0.005	[56]
Paper	Screen printing	Paper	Photolithography	5.0	/	−	N/A	GOx/PB/rGO−TEPA/SPEs	Chrono (E = −0.3 V)	0.1−25	7.18 μA mmol^−1^ L cm^−2^	0.025	[57]
PET	Screen printing	PET	UV laser−cutter	4.8	/	−	/	GOx/PB	Chrono (E = 0.0 V)	0.1−1.0	1.27 μA mmol^−1^ L cm^−2^	0.024	[21]
Filter paper	Stencil printing	Fabric	Wax printing	22.4	/	−	N/A	GOx	Amp (E = 0.4 V)	0.25−20	-	0.44	[27]
Ceramic	Screen printing	PDMS	Mold	7.7	/	−	N/A	CS−RGO−NiNPs	Amp (E = 0.6 V)	0.2−9.0	318.4 μA mmol^−1^ L cm^−2^	0.0041	[58]
Paper or polyester film	Screen printing	Polyester−cellulose paper	Photolithography/Wax printing	−	/	EUR 0.02	N/A	GOx	Chrono *	0−22.2	0.0043 μA mmol^−1^ L cm^−2^	0.22	[28]
Paper	Screen printing	−	Photolithography	5.0	/	−	N/A	GOx	Chrono (E = 0 V)	0−100	4.92 μA mmol^−1^ L cm^−2^	0.21 ± 0.02	[59]
Glossy paper (ArjoWiggins)	Inkjet printing	−	−	30.0	/	−	N/A	Nafion/GOx−Fc−Chi/PEDOT:PSS	Amp (E = 0.25 V)	0.025−0.9	-	-	[60]
Whatman paper	Wax printing	−	−	10.0	/	EUR 0.52	N/A	GOx−HRP/Ferro/C−ink	Chrono (E = −0.1 V)	0.3−15	16.14 μA mmol^−1^ L cm^−2^	0.12	[61]
Filter paper	Wax printing and Screen printing	−	−	25.0	/	EUR 0.07	N/A	GOx/4−APBA	Chrono (E = 0.2 V)	0.05−100	13.44 μA mmol^−1^ L cm^−2^	0.86	[62]
Nitrocellulose membrane	Screen printing	−	−	−	/	−	N/A	GOx/[Ru(NH_3_)_6_]^3+^	Chrono (E = 0.0 V)	0−27.7	12.75 μA mmol^−1^ L cm^−2^	−	[63]
Alumina	Screen printing	−	−	2.0	/	−	N/A	GOx/Glu/Ird	Amp (E = 0.25 V)	0−15	11.90 μA mmol^−1^ L cm^−2^	−	[64]
Indium Tin Oxide	Thermal inkjet printing		−	−	X	−	N/A	GOx/PEDOT:PSS/ITO	Chrono (E = 0.6 V)	0−60	6430 μA mmol^−1^ L cm^−2^	−	[65]

* means the publication did not mention about operating potential. N/A means not applicable. / means integrated three electrodes on the same platform. X means separated three electrodes or the used of external auxiliary electrode and reference electrode.

**Table 2:** Determination of glucose level in a serum sample measured by the MF-iGPE glucose biosensors.

Samples	Detected Value (mmol L^−1^)	Reference Value (mmol L^−1^)
Diabetes patient	9.6 ± 1.3 (*n* = 3)	10.05 *
Healthy subject	6.8 ± 0.8 (*n* = 3)	[1]

* Reference value provided by the hospital (standard hexokinase-spectrophotometric method for glucose measurement). ^1^ Reference values covering the healthy glucose level are cited from Anshun Zhao et al. [66], Yan Du et al. [67]

## Data Availability

The data presented in this study are available on request from the corresponding authors.

## Supplementary Materials

The following supporting information can be downloaded at: https://www.mdpi.com/article/10.3390/bios13040446/s1, Figure S1 Cyclic voltammograms of different track widths of GPE consisted of (A) 0.3, (B) 0.5, and (C) 1.0 mm.; Figure S2 SEM images at (A) low and (B) high magnitude of Prussian blue modified on iGPE. (C) EDX spectrum of PB modified on iGPE.; Figure S3 Cyclic voltammograms of PB/iGPE in the absent and present hydrogen peroxide containing 0.1 mol L^−1^ PBS and 0.1 mol L^−1^ KCl; Figure S4 Chronoamperograms of glucose in the presented of (A) ascorbic acid and (B) dopamine. (C) The relative current changed interpreted from chronoamperograms A and B; Figure S5 (A) Stability of the glucose sensor for different days, the response at the first day was defined as 100%. (B) Repeatability of the glucose sensor for several cycles, the response at the first time was defined as 100%. Table S1 The recovery values obtained from the spiked standard glucose in human serum sample.
