# Supplementary material for: Craft-and-Stick Xurographic Manufacturing of Integrated Microfluidic Electrochemical Sensing Platform
Source: Biosensors (Basel). 2023 Mar 31;13(4):446. doi: 10.3390/bios13040446 (PMC10136003; doi:10.3390/bios13040446)
Supplement: Supplementary file 1 [file biosensors-13-00446-s001.zip › biosensors-2157307-supplementary.pdf]

biosensors

MDPI

*Supplementary information*

# Craft-and-stick xurographic manufacturing of integrated microfluidic electrochemical sensing platform

**Supatinee Kongkaew[1,2,3,5], Lingyin Meng[1], Warakorn Limbut[2,3,5,*], Guozhen Liu[7], Proespichaya Kanatharana[2,3,4], Panote Thavarungkul[2,3,4], Wing Cheung Mak[1,6*]**

[1] Biosensors and Bioelectronics Centre, Division of Sensor and Actuator Systems, Department of Physics, Chemistry and Biology, Linköping University, SE-581 83 Linköping, Sweden
[2] Center of Excellence for Trace Analysis and Biosensor, Prince of Songkla University, Hat Yai, Songkhla 90112, Thailand
[3] Center of Excellence for Innovation in Chemistry, Faculty of Science, Prince of Songkla University, Hat Yai, Songkhla 90112, Thailand
[4] Division of Health and Applied Sciences, Faculty of Science, Prince of Songkla University, Hat Yai, Songkhla 90112, Thailand
[5] School of Medicine, The Chinese University of Hong Kong, Shenzhen 518172, China
[6] Division of Physical Science, Faculty of Science, Prince of Songkla University, Hat Yai, Songkhla 90112, Thailand
[7] Department of Biomedical Engineering, The Chinese University of Hong Kong, Hong Kong SAR, China
* Correspondence: warakorn.l@psu.ac.th (W.L.); wing.cheung.mak@cuhk.edu.hk (W.C.M.)

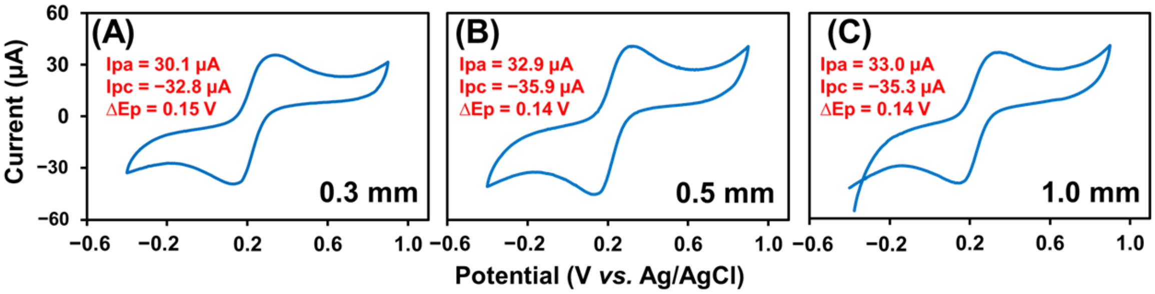


**Figure S1** Cyclic voltammograms of different track widths of GPE consisted of **(A)** 0.3, **(B)** 0.5, and **(C)** 1.0 mm.

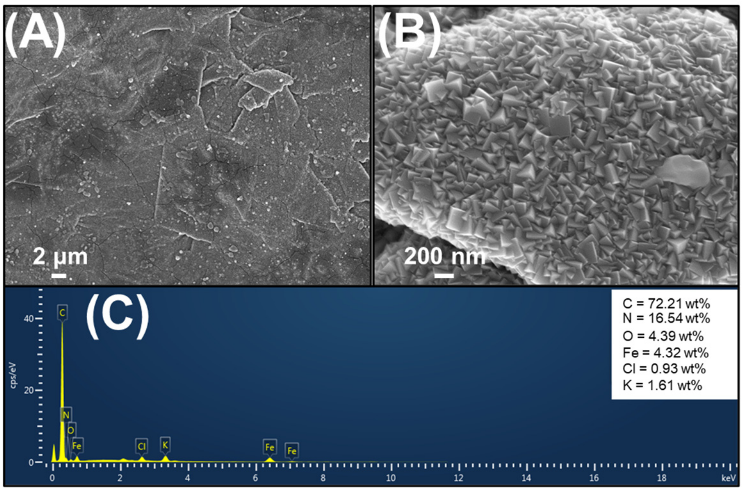


**Figure S2** SEM images at **(A)** low and **(B)** high magnitude of PB modified on iGPE. **(C)** EDX spectrum of PB modified on iGPE.

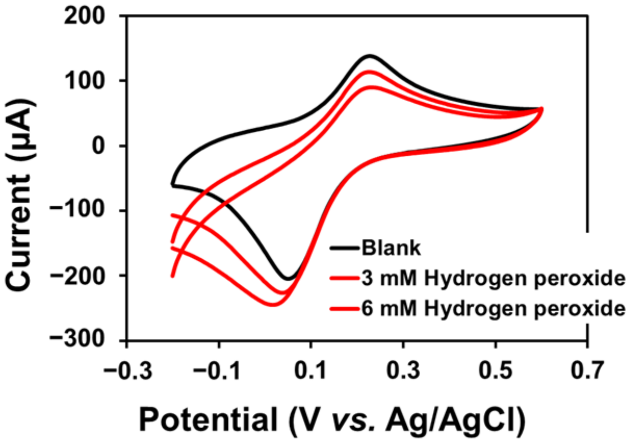


**Figure S3** Cyclic voltammograms of PB/iGPE in the absent and present hydrogen peroxide containing 0.1 mol $L^{-1}$ PBS and 0.1 mol $L^{-1}$ KCl.

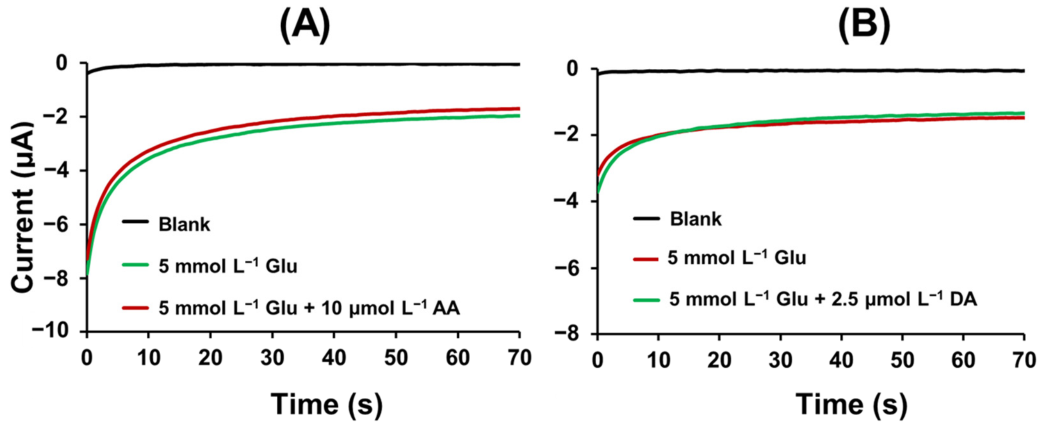


**(C)**

| Compounds | Relative current (%) | | | Average | SD |
|---|---|---|---|---|---|
| | 1 | 2 | 3 | | |
| 5 mmol L$^{-1}$ Glucose | 100 | 100 | 100 | 100 | - |
| 5 mmol L$^{-1}$ Glucose + 10 μmol L$^{-1}$ Ascorbic acid | 90.0 | 92.6 | 90.5 | 90.5 | 1.1 |
| 5 mmol L$^{-1}$ Glucose + 2.5 μmol L$^{-1}$ Dopamine | 94.2 | 88.2 | 91.8 | 91.8 | 2.5 |

**Figure S4** Chronoamperograms of glucose in the presented of **(A)** ascorbic acid and **(B)** dopamine**. (C)** The relative current changed interpreted from chronoamperograms A and B.

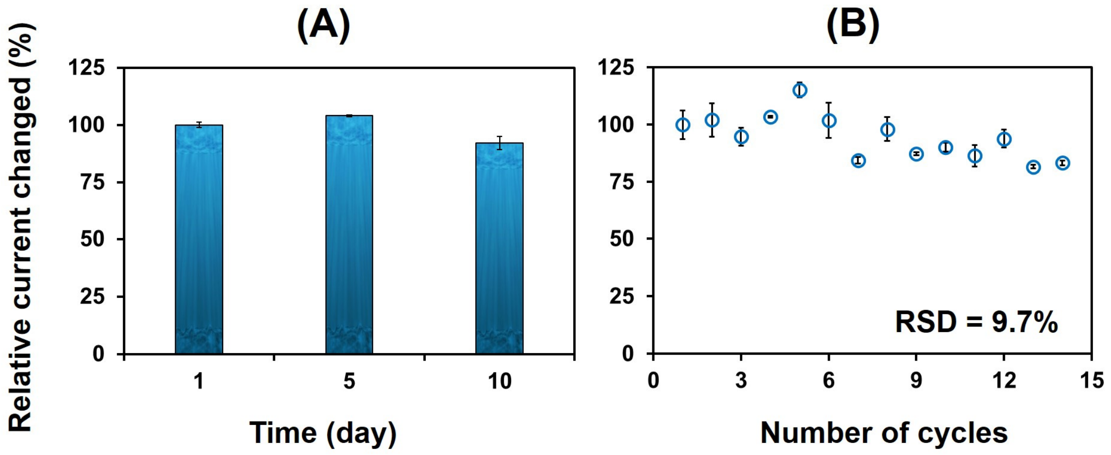


**Figure S5 (A)** Stability of the glucose sensor for different days with current responses to 5.0 mmol L$^{-1}$ glucose (n=3), the current response at the first day was normalized as 100%. **(B)** Repeatability of the glucose sensor for several cycles, the response at the first time was defined as 100%.

**Table S1** The recovery values obtained from the spiked standard glucose in human serum sample

| Concentration of glucose (mmol $L^{-1}$) | | Recovery (%) |
|---|---|---|
| Spiked | Found | |
| 0 | 0.96 | - |
| 1 | 2.05 | 108.8 |
| 2 | 2.72 | 88.2 |
| 3 | 3.62 | 88.7 |
